# Typical everyday movements cause specific patterns in heart rate

**DOI:** 10.3389/fphys.2024.1379739

**Published:** 2024-07-26

**Authors:** Max J. Heidelbach, Dirk Cysarz, Friedrich Edelhäuser

**Affiliations:** Integrated Curriculum for Anthroposophic Medicine, University of Witten/Herdecke, Witten, Germany

**Keywords:** light physical activity, heart rate regulation, heart rate pattern, transient oscillation, everyday movements, heart rate variability, cardiovascular health, public health

## Abstract

Physical inactivity and sedentary behaviour are important risk factors for cardiovascular disease. Knowledge about the impact of everyday movements on cardiac autonomic regulation is sparse. This study aims to provide evidence that typical everyday movements show a clear impact on heart rate regulation. 40 healthy participants performed two everyday movements: (1) calmly kneeling down (“tie one’s shoes”) and standing up again and (2) raising the arms to the horizontal (“expressive yawning”). Both movements elicited reproducible pattern in the sequence of heart periods. Local minima and local maxima appeared in the transient period of approx. 30 s. The regulatory response for ergometer cycling, which was used as control, did not show a pattern formation. Calmly performed everyday movements are able to elicit rich cardiac regulatory responses including specific patterns in heart rate. These newly described patterns have multiple implications for clinical and rehabilitative medicine, basic research, digital health data processing, and public health. If carried out regularly these regulatory responses may help to mitigate the burden of physical inactivity and enrich cardiovascular regulation.

## Introduction

Physical inactivity and obesity are major risk factors for cardiovascular disease (CVD) (Lee et al., 2012; Lear et al., 2017; Ahmadi et al., 2022; Liang et al., 2022) and CVD causes most deaths worldwide, with an increasing incidence (Roth et al., 2020). Obesity is a growing problem worldwide, with its incidence having almost tripled since the 1970s (NCD Risk Factor Collaboration NCD-RisC, 2017). Simultaneously, physical activity has decreased (Guthold et al., 2018; Stockwell et al., 2021; World Health Organization, 2022), especially during the COVID-19 pandemic, which was caused by lockdowns or restrictions on meeting other people (Runacres et al., 2021). It is well known that physical activity is an essential factor in preserving and restoring cardiac and general health. Hence, physical activity has a positive effect on public health (Lear et al., 2017). In general, physical activity is part of daily life and, for example, the higher the number of steps per day, the lower the all-cause and cardiovascular mortality (Banach et al., 2023).

Everyday movements are rarely considered relevant for cardiac health because they are obviously not considered as an exercise. However, recent findings suggest that even light-intensity activity is more beneficial than no activity (Del Pozo Cruz et al., 2021; World Health Organization, 2022). Changing sedentary behaviour to any kind of simple physical activity, even standing, seems to be favourable (Blodgett et al., 2024).

However, knowledge about the impact of simple everyday movements on cardiac autonomic regulation is sparse. Generally, there is a wide range of typical everyday movements, for example, getting up from a chair, walking a few steps and sitting down again; stretching the arms during sitting. Each movement involves activity of skeletal muscles and even muscular activity at low workloads has been shown to have an impact on cardiovascular regulation, e.g., heart rate and stroke volume (Mortensen et al., 2013). Hence, such movements should be considered relevant to cardiac autonomic regulation and their impact on health maintenance and recovery should be properly assessed.

Transitions in cardiac autonomic regulation between different states like, e.g., standing and movement have rarely been addressed. Such transitions can be used as an indicator of the regulatory capacity of cardiac autonomic regulation. In a recent study, we have shown that the repetition of a slowly squatting and standing up again induced very low frequency oscillations in heart rate variability (HRV) (Edelhäuser et al., 2015). I.e., the frequency of squatting elicited the very low frequency oscillations showing that the movement was responsible for the oscillatory pattern in the heart rate. Here, we aim to show that everyday movements have a clear immediate impact on cardiac autonomic regulation particularly during the beginning of a movement. Having considered a variety of everyday movements, we selected the following two body movements carried out predominantly in either a vertical or a horizontal direction: (1) kneeling down on one knee as if, for example, to tie shoelaces, and (2) raising the arms and moving them horizontally as if stretching during expressive yawning. Ergometer cycling, which is considered a healthy exercise (Kelly et al., 2014), was used as a control. We show that only the two everyday movements elicited distinct, yet reproducible, transition patterns of cardiac autonomic regulation as captured by the series of heart periods (i.e., the RR interval series).

## Results

**FIGURE 1:**
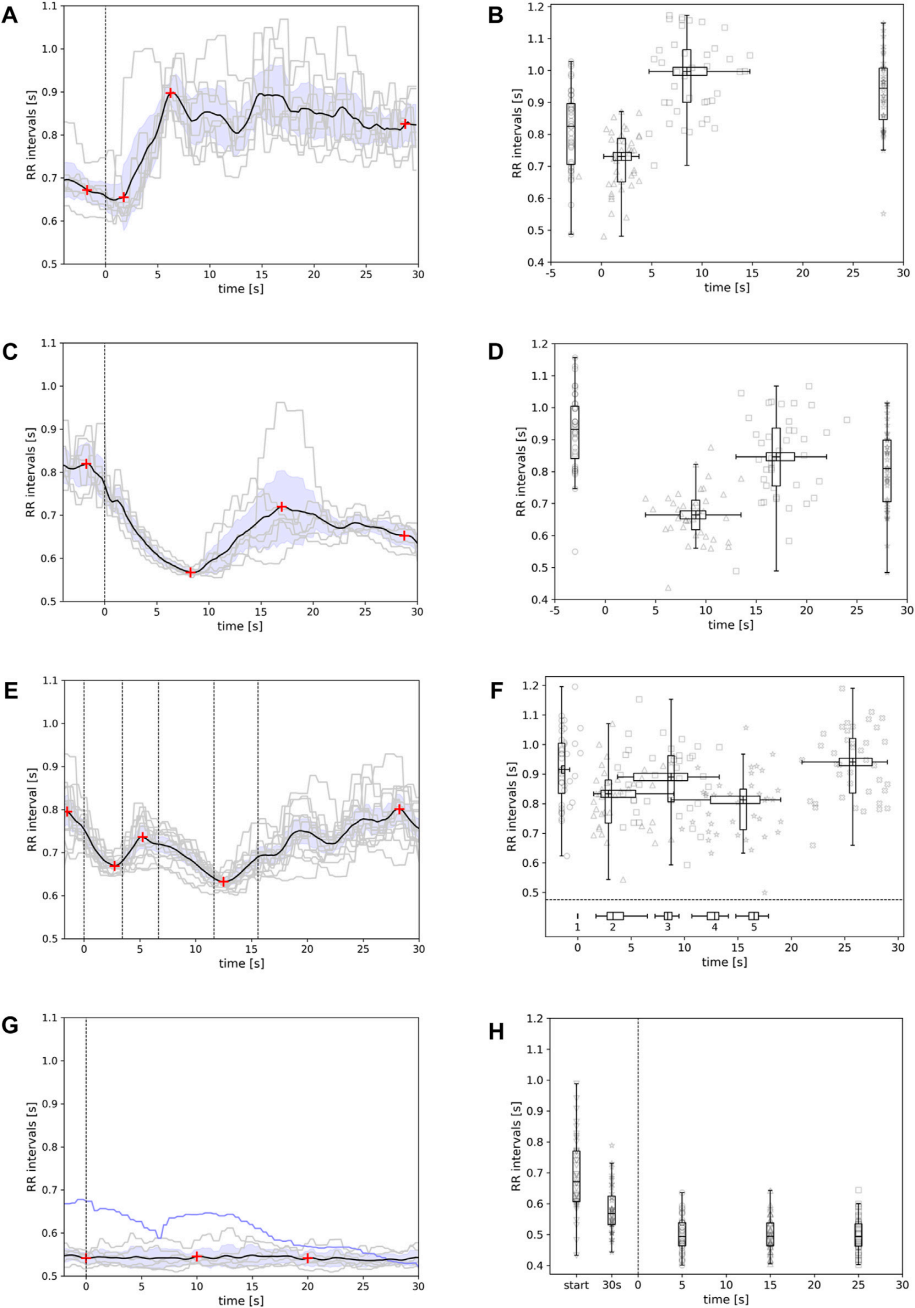
Example of patterns in the RR interval series during **(A)** kneeling down, **(C)** getting up again, **(E)** moving the arms and hands from the lap to a straight horizontal line at shoulder level, and **(G)** cycling on an ergometer (blue line: regulation within first 30 s of cycling). Grey lines depict the RR interval series during each repetition of the movement (8 repetitions); the black line shows the average RR interval pattern. The vertical dashed line in **(A)** and **(C)** indicates the start of the movement. In **(E)**, the vertical dashed lines from left to right indicate the 1) start of raising arms, 2) start of moving arms outwards, 3) start of moving arms inwards, 4) start of lowering arms to the lap, and 5) end of arm movement. The red crosses indicate the local minima/maxima or time points [in **(G)**] used to quantify the pattern of each average RR interval series. The results of the pattern quantification for all participants are shown in **(B)** – kneeling down, **(D)** – getting up, **(F)** – arm movement, and **(H)** – ergometer cycling (including at start and after 30 s of cycling). For further details, see text.

The RR interval time series during kneeling down (exercise 1a) showed a clear pattern formation in all participants. The observed pattern was reproducible across all eight repetitions. A local minimum appeared in the RR intervals shortly after the movement started (Figure 1A, black line). This expresses a shorter RR interval and therefore an increased heart rate. After 5 s, the RR intervals increased, and a local maximum appeared, showing a lower heart rate. After about 20 s of kneeling, the RR intervals slightly decreased again. Hence, four RR intervals were used to quantify this transient oscillation seen in kneeling down (Figure 1A, red crosses): (1) RR interval during standing (i.e. 2 s before the movement started); (2) local minimum RR interval after 2–5 s; (3) local maximum RR interval after 8–12 s; and (4) RR interval after 28 s in the kneeling position.

The pattern in RR interval during kneeling down was reproducible across all eight repetitions in one participant (Figure 1A). The entire group also showed this pattern. Figure 1B shows boxplots of the RR intervals before starting the movement (median: 825 ms, 72.2 bpm, cf. Table 1), during the minimum (median: 731 ms, 82.1 bpm; occurring 2 s after start), during the maximum (median: 997 ms, 60.2 bpm; occurring 8.5 s after start), and at the end (median: 944 ms, 63.5 bpm). There were variations between the distributions (*p* < 0.001), and the pairwise comparisons showed differences between the distributions (*p* < 0.001), except for the distributions of the local maximum and interval at the end. The distributions of the times of the local minimum and the local maximum (Figure 1B, horizontal distributions) also differed (*p* < 0.001).

**TABLE 1:** Quantification of patterns in the RR interval series.

Kneeling down	RR pre [ms]	RR min [ms]	RR max [ms]	RR post [ms]	
Median*	825	731^a^	997^a,b^	944^a,b^	
IQR	706–896	651–787	900–1,065	847–1,007	
	**t pre [s]**	**t min [s]**	**t max [s]**	**t post [s]**	
Median*	−2	2·0	8·5	28	
IQR	··	1·2–3·0	7·1–10·5	··	
**Getting up**	**RR pre [ms]**	**RR min [ms]**	**RR max [ms]**	**RR post [ms]**	
Median*	932	664^a^	846^b^	809^a,b,c^	
IQR	841–1,004	618–711	754–936	706–898	
	**t pre [s]**	**t min [s]**	**t max [s]**	**t post [s]**	
Median*	−2	9·0^e^	17·0^e^	28	
IQR	··	7·4–10·0	16·0–18·8	··	
**Raising arms**	**RR pre [ms]**	**RR min1 [ms]**	**RR max1 [ms]**	**RR min2 [ms]**	**RR max2 [ms]**
Median*	915	833^a^	890^b^	813^a,c^	941^b,c,d^
IQR	835–1,004	735–880	807–962	712–849	836–1,021
	**t pre [s]**	**t min1 [s]**	**t max1 [s]**	**t min2 [s]**	**t max2 [s]**
Median*	−2	2·9	8·8	15·5	25·8
IQR	··	2·2–5·4	5·3–10·3	12·4–17·1	24·5–27·6
**Ergometer cycling**	**RR start [ms]**	**RR 30s [ms]**	**RR t5s [ms]**	**RR t15s [ms]**	**RR t25s [ms]**
Median*	671	568	493	495	493
IQR	606–771	532–624	464–539	463–583	463–535

*Friedman test *p* < 0·001, ^a^ *p* < 0·001 vs. 1st col., ^b^ *p* < 0·001 vs. 2nd col., ^c^ *p* < 0·01 vs. 3rd col., ^d^ *p* < 0·001 vs. 4th col., ^e^ *p* < 0·001 vs. corresponding time during kneeling down.

RR intervals are visible before (RR pre), within as a minimum and maximum (RR min and RR max) and after (RR post) the exercise in milliseconds. Times t pre, t min, t max and t post refer to the time before the start of the movement (pre), time of maximum, time of minimum and time after the transition (post).

A different pattern was observed during getting up after kneeling (Figure 1C, black line). The RR intervals decreased during getting up and subsequently increased again. After 25 s of standing, the RR intervals slightly decreased again. Hence, a local minimum and a local maximum appeared. Four different RR intervals were again used to quantify the pattern (Figure 1C, red crosses): (1) RR interval during kneeling (i.e. 2 s before getting up); (2) local minimum RR interval after approximately 10 s; (3) local maximum RR interval after approximately 15–20 s; and (4) RR interval after 28 s in the standing posture.

Characteristic local minimum and maximum were observed in all 40 participants. Figure 1D shows the boxplot of the RR intervals before getting up (932 ms, 64.4 bpm, cf. Table 1), during the minimum (664 ms, 90.3 bpm; occurring 9 s after start), during the maximum (846 ms, 70.9 bpm; occurring 17 s after start), and at the end (809 ms, 74.1 bpm). There were variations between the distributions (*p* < 0.001). The pairwise comparisons showed differences between the distributions (*p* < 0.001), except for those at the start (during kneeling) and during the maximum. The distributions of the times of the local minimum and the local maximum (Figure 1D, horizontal distributions) differed (*p* < 0.001).

Raising the arms to shoulder height also showed a reproducible and specific pattern in the RR interval series (Figure 1E, black line). When raising the arms, the RR intervals decreased whereas during outward movement of the arms the RR intervals increased; and moving the arms inwards and lowering them decreased the RR intervals. After the end of the movement, the RR intervals increased again. Hence, two local minima and two local maxima appeared (Figure 1E, red crosses). The RR interval 2 s before the movement started and the sequence of the four local minima and maxima were used to quantify the pattern.

Figure 1F shows the boxplots of the RR intervals before starting the movement (median: 915 ms, 65.6 bpm), during the first local minimum (median: 833 ms, 72 bpm), during the first local maximum (median: 890 ms, 67.4 bpm), during the second minimum (median: 813 ms, 73.8 bpm), and during the maximum at the end (median: 941 ms, 63.8 bpm). There were variations between the distributions (*p* < 0.001). The pairwise comparisons showed differences between the distributions in consecutive order (*p* < 0.001). The RR intervals before the start and during the second minimum differed from one another, and the RR intervals at the end differed from the RR intervals both during the first minimum and during the maximum (*p* < 0.001). The distributions of the times of the two local minima and two local maxima (Figure 1F, horizontal distributions) significantly differed from each other (*p* < 0.001). The boxplots below the dashed lines indicate the interindividual variations in the timing of starting the different parts of the arm movement (1: raising arms, 2: moving arms outwards, 3: moving arms inwards, 4: moving arms downwards, 5: arms on lap).

Finally, we tested patterns in the RR interval series during standard ergometer training. During the transitional period following the start of cycling (30 s), a decrease in the RR intervals was observed (cf. Fog. 1g, blue line and Figure 1H; cf. boxplots left of the vertical dashed line; median: 671 ms, 89.4 bpm at start; 568 ms, 105.6 bpm after 30 s). After this initial decrease in the RR intervals, there were no more changes in the RR intervals (cf. Figure 1G, black line; Figure 1H boxplots right of the vertical dashed line; median: 493 ms, 121.7 bpm at 5, 15, and 25 s). There was variation between the distributions (*p* < 0.001). The RR interval distributions at points during the starting period (0 and 30 s from start) differed from each other (*p* < 0.05) and from the three distributions after the starting period (*p* < 0.001).

## Discussion

The lack of physical activity during, e.g., sedentary behaviour is considered a mortality risk factor for CVD and contributes to an unhealthy lifestyle (Stamatakis et al., 2019). Here, we show that even simple everyday movements have a clear impact on cardiac autonomic regulation and hence may counteract the burden of sedentary behaviour.

The simple everyday movements investigated in this study invoked clear transition patterns in the heart period series. If carried out calmly the series of heart periods showed a clear transient oscillation. I.e., the cardiac autonomic regulation showed a specific oscillation and needed approx. 30 s to adapt to the physiological changes caused by the movement. The pattern associated with each movement sequence was replicable intra- and interindividually. In this study, kneeling down consisted of one knee in the kneeling position and one knee in a squatting position. Hence, the present results can be compared to previous reports on cardiovascular changes during squatting. In fact, the alterations in cardiac autonomic regulation are similar to the alterations found during squatting (Rossberg and Penaz, 1988; Edelhäuser et al., 2015). In particular, a comparison with heart transplant patients showed that the circulatory responses during squatting could be primarily attributed by increased venous return to the heart independent of the functioning of the autonomic nervous system (Hanson et al., 1995). Simultaneously, cardiac output increases because the increased blood volume at the heart must be carried back to the periphery (Sharpley-Shafer, 1956; O'Donnell and McIlroy, 1962; Lance and Spodick, 1977). Total peripheral resistance is not altered during squatting (O'Donnell and McIlroy, 1962; Krediet et al., 2005). These alterations lead to an increase in blood pressure that help to mitigate symptoms of, e.g., orthostatic intolerance (van Lieshout et al., 1992; Krediet et al., 2005). Furthermore, resting in a squatting position is healthier compared to resting in a sitting position (Raichlen et al., 2020) probably as a consequence of the adaptations in cardiac autonomic regulation during squatting. The adaptations to these alterations obviously need several seconds and, hence, lead to a specific pattern formation in heart rate during the transition between standing and squatting.

Interestingly, the minimum in the pattern during kneeling down appears faster compared to the minimum in the pattern during getting up. The minimum of RR intervals during kneeling down may reflect the heart response on the immediate pooling of blood caused by the increased venous return: the heart quickly beats faster. During getting up the venous return decreases and consequently the heart has to beat faster to keep blood pressure constant. This response takes a few seconds longer indicating physical limits of blood flow.

To the best of our knowledge, structured arm movements have not yet been investigated with respect to cardiovascular changes. Similar to kneeling movements, structured arm movements produce a typical and reproducible pattern in the RR interval series. The impact of arm movements at low workloads on cardiac autonomic regulation is similar to leg movements (Bevegård et al., 1966; Astrand, 1971; Boushel et al., 2014; Calbet et al., 2015). Hence, cardiac output is increased during arm movements according to the oxygen demand of the arm muscles (Couser et al., 1992; Mortensen et al., 2013). Furthermore, an increase in gastric pressure induced by the raised arm positions should be considered (Couser et al., 1992). These pressure changes also may lead to differences in venous return to the heart and also impact the timing of inspiration and expiration, which consequently influences respiratory sinus arrhythmia (Niizeki et al., 1993; Fisher et al., 2015). As a consequence of the adaptation to these changes, a pattern formation appears in cardiac autonomic regulation.

Although respiration was not recorded in this study, it was observed that the start of movement was often aligned to expiration (kneeling down) or inspiration (standing up). The arm movements were also often aligned to inspiration. Recent findings suggest that arm movements and breathing can be coordinated if the temporal conditions are set appropriately (Ebert et al., 2000). Regardless of the coordination, breathing patterns during the movement led to respiratory sinus arrhythmia in the present study (cf. grey lines in Figures 1A, E). The repetitions of each movement were matched to get an average response that is averages out the respiratory influence to a certain degree. The coordination between everyday movements and respiratory timings (inspiration, expiration) and its impact on respiratory sinus arrhythmia needs to be clarified in studies focussing on respiratory patterns.

Ergometer cycling at 60–80 W does not show a pattern formation in the series of heart periods during the transitional period after cycling started. This can be attributed to the continuous movement in contrast to the repeatedly performed movements described above. Furthermore, the steady state during ergometer cycling also did not cause any further response in cardiac autonomic regulation as indicated by the steady state in the series of heart periods (Lunt et al., 2011).

The pattern formation in the heart period series in healthy subjects indicates a slow response to the everyday movements of the order of 30 s. The movements that drive these responses should therefore be carried out calmly to allow the full cardiac autonomic regulatory response to develop. Generally, slowly performed everyday movements should also lead to transition patterns of heart rate in, e.g., obese adults because the functional aspects stated above still apply. However, obese adults show an increase in intravascular blood volume and filling pressures in heart cavities along with an increase in cardiac output, systemic vascular resistance and heart rate (Koliaki et al., 2019; Koenen et al., 2021). The increase in heart rate implies a reduction of heart rate variability and, hence, the resulting transition pattern in heart rate is likely less pronounced compared to healthy subjects. Regardless of these alterations, an increase in physical activity still has positive effects on cardiac autonomic regulation (Kaikkonen et al., 2014).

The range of the heart periods seen during short segments of 30–60 s in duration while performing everyday movements could be used to quantify the regulatory capacity in individuals and in particular patients. This capacity could be considered an indicator of cardiac regulatory health and could, for example, be tracked by wearable devices that quantify heart rate variability (HRV). This approach could be used to understand the health impact of individual lifestyles with regard to movement. Personalised recommendations to improve individual cardiac regulatory capacity could subsequently be given (Natarajan et al., 2020; Negreiros et al., 2022). Furthermore, AI could be used to find distinct pattern formation in RR interval series in relation to other health data.

The intensity of many everyday movements should be considered at the lower range of light-intensity physical activity (Herrmann et al., 2024) because these movements do not increase the heart rate, as oxygen demand in the affected muscles does not increase. Hence, the energy expenditure involved should be considered to be low. Nevertheless, from the perspective of cardiac autonomic regulation, the impact of everyday movements is far greater than that of, for example, cycling at a constant speed.

As the energy expenditure involved in many everyday movements is low (Herrmann et al., 2024), cardiac autonomic regulation could be improved even in individuals with minimal physical capacity, such as obese or chronically ill patients (Kimm et al., 2005; Conn et al., 2008). Everyday movements such as the arm movement studied herein could also be performed by patients using wheelchairs. In general, everyday movements can be used to promote physical activity at a very basal level (Haseler et al., 2019).

Cardiac autonomic regulatory capacity could be trained with properly and regularly performed everyday movements (Lin et al., 2022). This is of clinical importance in terms of the restoration of cardiac autonomic regulation and heart rate variability (HRV) after, for example, myocardial infarction (Fei et al., 1996) or in patients with coronary artery disease (Osterhues et al., 1997). Especially the regulatory capacity in terms of the transient oscillation seems to be relevant in this context (Bauer et al., 2006). Furthermore, such transient oscillations could be partially responsible for the increase in very low oscillations during physical activity (Bernardi et al., 1996)^1^. Hence, we suggest that everyday movements be considered for inclusion also for rehabilitation purposes. These movements and their frequent repetition can be easily integrated into daily activities. Our study suggests that even slow light-intensity physical activity shows an instantaneous influence on cardiac autonomic regulation and therefore fosters a healthier condition. However, the long-term impact of regular (slow) everyday movements on the transient patterns in the heart period series needs to be investigated. Taken together, in healthy subjects each instance of slow light-intensity physical activity represented by an everyday movement leads to a specific response pattern in heart rate, i.e., drives the heart rate regulation in a particular way.

## Methods

### Study design and participants

Forty healthy adults (age range: 18–65 years, 22 women) were enrolled in the study. Individuals with a healthy condition and a body mass index of 18–25 kg/m^2^ were included, although those older than 40 years with a slightly higher body mass index (up to 28 kg/m^2^) were also included. Individuals taking long-term medication (except oral contraceptives) or experiencing CVD such as hypertension, hypotension, or heart failure; diabetes mellitus; thyroid gland diseases; back, knee, or hip pain or any other movement-limiting disease; or an acute infection including fever were excluded.

### Experimental protocol

**FIGURE 2:**
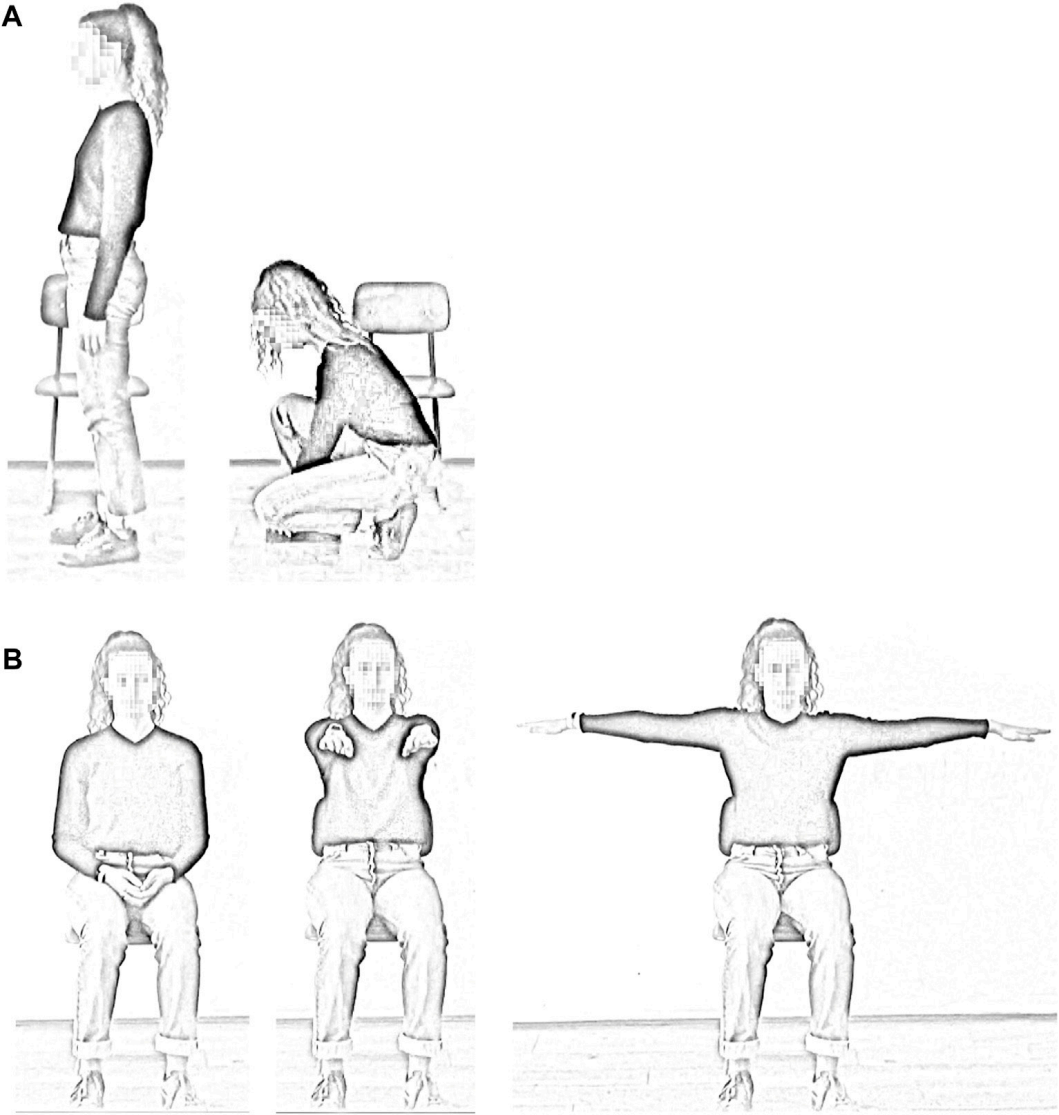
Examples of the movements. Kneeling down: the subject stands for 30 s and then kneels down for 30 s **(A)**. Arm movement: the arm movement begins with the hands resting on the lap. The hands are then raised forward to shoulder height and then moved outwards to form a horizontal line. The arms are moved forward again and then placed on the lap **(B)**.

Movements were performed in groups consisting of five individuals each. The movements were synchronised using video and metronomic guidance by the study representative. The first movement sequence entailed kneeling down on one knee and place the hands on the foot of the other leg (“tie one’s shoes”; exercise 1a, see Figure 2A) and standing up again (exercise 1b), each performed over 30 s. Hence, a full sequence of kneeling down and standing up again took 1 min. The two parts of the sequence were analysed separately. The second movement sequence entailed raising the arms while in an upright sitting position (“expressive yawning”; exercise 2, see Figure 2B) as follows: (1) The arms and hands, pointing forwards, were raised from the lap to shoulder level and (2) then moved outwards to form a straight horizontal line. Next, (3) the arms were moved back to the forward position and (4) then moved down to the lap again. Each part of the movement was performed over 5 s.

Cycling on an ergometer bicycle was performed as a control (exercise 3). The required cadence was 50–60 rpm, while the required workload was 60 W for the female participants and 80 W for the male participants.

Each exercise was performed for 8 min, i.e., the exercise was repeated several times. Between the 8-min periods, the participants rested for 2 min, quietly sitting on chairs. At the start and end of the procedure, there was also 5 min of quiet sitting. The participants were given no restrictions with respect to breathing. They were asked to perform the exercises quietly.

### Measurements

Each participant was equipped with a 1-channel ECG-recording device (Faros 180, Bittium Corp., Finland) on the chest. The ECG was sampled at 250 Hz. The device also recorded movements using an accelerometer. The accelerometer signal was sampled at 100 Hz. A second device was placed on the right wrist to record arm movements. The accelerometer time series of both devices were synchronized using a marked signal in the accelerometer data obtained during a jump on the spot.

The times of the R-peaks in the ECG were automatically detected by the device. The data were checked for artefacts and manually corrected when necessary (<0.1% of all R-peaks). The series of differences between the times of successive R-peaks formed the RR tachogram, i.e., the series of instantaneous heart periods. The RR tachogram was re-sampled at 4 Hz to form a proper RR time series.

### Heart period patterns

Patterns in the heart period series triggered by the movement sequences were analysed as follows. Each repetition of the movement sequence had to be identified. For exercise 1, the initiation of the kneeling and rising movements was separately determined by a clear change in the accelerometer data using a changepoint detection algorithm. These changes could be identified in all subjects for each repetition of the movement and, hence, each repetition of the movement could be included in the analysis. A 30-s sequence of the RR time series was used for the analysis starting 5 s before the initiation of the movement. The repetitions of the arm movement (exercise 2) were also identified according to clear changes in the accelerometer data using changepoint detection. Next, the average RR time series across all eight repetitions was calculated for kneeling down (cf. Figure 1A), getting up (cf. Figure 1C) and the arm movement (cf. Figure 1E).

Ergometer cycling did not show clear changes in the accelerometer data and, hence, the time series had to be partitioned into pre-defined sequences. A particular pattern in the RR interval series did not occur (cf. Figure 1G). To account for a response in cardiac activity caused by the start of cycling, we calculated the average RR interval at the start and after 30 s. To assess the dynamics of the RR interval series analogously to those seen in the other exercises, we split the RR interval series into eight successive 30-s series and calculated the average RR intervals at 0, 10, and 20 s.

### Statistical procedures

Differences in the RR intervals of the four (kneeling down, getting up) and five (arm movement, ergometer cycling) time points were assessed using a non-parametric analysis of variance for repeated measurements (Friedman test). The Conover test (including adjustments for multiple comparisons) was used for pairwise comparisons in case of significant differences. *p* < 0.05 was considered statistically significant.

## Data Availability

The raw data supporting the conclusions of this article will be made available by the authors upon request, without undue reservation.

## Footnotes

1 Physical activity seems to lead to non-stationarities in the heart period series that alter standard HRV parameters (Magagnin et al., 2011). HRV parameters, e.g., from binary symbolic dynamics analysis are still capable of quantifying HRV during non-stationarities appropriately (Spellenberg et al., 2020).
